# Maternal phthalate exposure, gestational length, and preterm birth risk: a prospective cohort study nested within a randomised trial

**DOI:** 10.1186/s12884-025-07980-8

**Published:** 2025-08-08

**Authors:** Karen P Best, Lisa N Yelland, Liu Ge, Zumin Shi, Shalem Leemaqz, Robert Gibson, Maria Makrides, Philippa Middleton

**Affiliations:** 1 SAHMRI Women and Kids, South Australian Health and Medical Research Institute, North Adelaide, South Australia; 2 Adelaide Medical School, The University of Adelaide, Adelaide, South Australia Australia; 3 School of Public Health, The University of Adelaide, Adelaide, South Australia Australia; 4 Department of Nutrition Sciences, College of Health Sciences, Qatar University, Doha, Qatar; 5 School of Agriculture Food and Wine, The University of Adelaide, Adelaide, South Australia Australia

**Keywords:** Pregnancy, Exposure, Phthalates, Environment, Prematurity, Endocrine disrupting chemicals, Prenatal

## Abstract

**Background:**

Preterm birth (< 37 weeks gestation) is a leading cause of infant morbidity and mortality, yet the underlying causes remain unknown in many cases. Environmental exposures, including endocrine-disrupting chemicals such as phthalates, have been implicated in preterm birth risk. Phthalates are commonly used as plasticisers in consumer products, resulting in widespread human exposure. While some studies suggest an association between maternal phthalate exposure and reduced gestational length, findings remain inconsistent. This study aimed to investigate the relationship between urinary phthalate metabolite concentrations and gestational length in an Australian pregnancy cohort.

**Methods:**

This prospective cohort study was nested within the Omega-3 to Reduce the Incidence of Prematurity (ORIP) trial. A total of 605 women with singleton pregnancies from South Australia provided urine samples between 22- and 26-weeks’ gestation for phthalate metabolite analysis. Thirteen phthalate metabolites were quantified using liquid chromatography-tandem mass spectrometry. Gestational age at birth was determined from medical records. Linear regression models assessed associations between phthalate concentrations and gestational length, adjusting for maternal characteristics including age, BMI, socioeconomic status, education, smoking, and alcohol consumption.

**Results:**

Phthalate metabolites were detected in > 99% of urine samples, with the highest concentrations observed for mono-ethyl phthalate (MEP), mono-isobutyl phthalate (MiBP), and mono-butyl phthalate (MBP). There was no evidence of an association between phthalate exposure and gestational length in either unadjusted or adjusted analyses. No significant association was found between phthalate exposure and preterm birth risk.

**Conclusions:**

Despite widespread phthalate exposure, no clear link was identified between maternal phthalate levels and shortened gestation in this Australian cohort. However, continued surveillance is needed to monitor emerging plasticiser exposures and inform public health policies on maternal and infant health.

**Trial registration number:**

Australian New Zealand Clinical Trials Registry number, ACTRN12613001142729. **Date of registration**: 27/09/2013.

**Supplementary Information:**

The online version contains supplementary material available at 10.1186/s12884-025-07980-8.

## Introduction

Preterm birth, defined as birth before 37 weeks of gestation, remains a major global health challenge, affecting an estimated 13.4 million infants annually and ranking as the leading cause of mortality in children under five [1, 2]. Survivors may face an increased lifetime risk of disability, including cerebral palsy, blindness, deafness, and behavioural and learning difficulties at school age, significantly impacting quality of life [3].

While some risk factors for preterm birth have been identified, the cause remains unknown in approximately two-thirds of cases, underscoring the need to explore potentially modifiable contributors to inform prevention strategies [4, 5]. Environmental exposures, such as phthalate esters are increasingly implicated in the timing of birth [6]. Phthalates are endocrine-disrupting chemicals used as plasticisers in consumer products such as food packaging, personal care items, and medical equipment [7–11]. Human exposure occurs through ingestion, inhalation, and dermal absorption, and is of particular concern in pregnancy due to potential effects on fetal development and maternal health [12]. Following exposure, these phthalate diesters are metabolised in the body into monoester and oxidative metabolites, which are excreted in urine and serve as reliable biomarkers of phthalate exposure. See common metabolites and typical exposure sources in Supplementary Table 1, Additional File 1.

Phthalate exposure among pregnant women is widespread, with several studies reporting that 90–100% of early pregnancy urine samples contain detectable phthalate metabolites [13–16]. While emerging evidence suggests that higher maternal phthalate exposure may be linked to shortened gestation and preterm birth, findings remain inconsistent [17–20]. Discrepancies across studies are likely attributed to differences in study design, population characteristics, and exposure levels. A key factor influencing these variations is regional differences in phthalate exposure, driven by differences in regulatory policies, product composition, consumer behaviours, and sociodemographic characteristics [21–23]. For example, dietary habits and personal care product use differ across population groups, influencing both the types and quantities of phthalates individuals are exposed to.

Most research on phthalate exposure during pregnancy has been conducted in the United States, Europe, and Asia. While Australia shares similarities with other industrialised countries, region-specific data are needed to understand how unique sociodemographic and environmental factors influence phthalate exposure and potential health effects. Such data can complement international research, strengthen public health messaging, and contribute to a more comprehensive understanding of the global impact of phthalates on maternal and infant health.

Addressing this gap is essential for informing policy decisions aimed at reducing environmental exposures and improving pregnancy outcomes worldwide. This study investigates the association between urinary phthalate metabolite concentrations, gestational length, and preterm birth in an Australian pregnancy cohort. Additionally, we examine how maternal characteristics, including sociodemographic and lifestyle factors, influence phthalate exposure levels.

### Participants and methods

This is a prospective cohort study, nested within the multi-centre, parallel-group randomised, blinded, controlled ‘Omega-3 to Reduce the Incidence of Prematurity’ (ORIP) Trial (Australian New Zealand Clinical Trials Registry number, ACTRN12613001142729). Full details of the ORIP trial methodology, results and statistical analysis plan are published elsewhere [24, 25]. In brief, 5,544 women with either a singleton or multiple pregnancy attending one of six centres across four states in Australia were recruited at their first antenatal appointment (< 20 weeks gestation), between 1 st November 2013 and 26th April 2017. Upon written consent, women were randomised to receive either omega-3 supplements [800 mg docosahexaenoic acid (DHA) and 100 mg eicosapentaenoic acid (EPA)/day] or isocaloric vegetable oil control capsules with trace fish oil for masking until 34 weeks of gestation. The primary outcome was early preterm birth, defined as birth before 34 weeks of gestation. Secondary outcomes included preterm birth, defined as birth before 37 weeks of gestation, and length of gestation [24]. Women who enrolled from two participating South Australian centres (Women’s and Children’s Hospital and Flinders Medical Centre) were approached to participate in a sub study which involved collection of urine samples for this nested cohort study. Both centres were coordinated by the same research team using standardised recruitment and data collection protocols, enhancing consistency across sites and supporting the generalisability of findings within the South Australian context. Human ethics approval was granted by the Women’s and Children’s Health Network Human Research Ethics Committee (HREC/16/WCHN/80). This manuscript adheres to the Strengthening the Reporting of Observational Studies in Epidemiology (STROBE) guidelines for cohort studies.

### Data and sample collection

At study enrolment, women were interviewed to obtain baseline clinical and sociodemographic details, including ethnicity, parity, education, smoking (at enrolment and within three months prior to pregnancy) and alcohol use (at enrolment). Height and weight measurements were collected by study staff at the enrolment visit. Pregnancy and birth data were extracted from maternal medical records by trained study staff independent of the clinical care team. Gestational length was determined using both the date of the last menstrual period and ultrasonographic data obtained in early pregnancy. Women attended a mid-trimester clinic visit between 22- and 26-weeks’ gestation where they provided a single urine sample allowing for quantification of phthalate monoester metabolites. Samples were stored at −80 °C until analysis. Urinary phthalate metabolites, which are highly stable in samples stored at temperatures ≤ 20 °C, are the preferred biomarker for assessing phthalate exposure [26, 27]. While phthalate-free collection containers were not used, the measurement of metabolites (rather than parent compounds) substantially reduces the risk of external contamination.

### Phthalate exposure

In this study thirteen phthalate metabolites were measured in each maternal urine sample; mono-methyl phthalate (MMP); mono-ethyl phthalate (MEP); mono-isobutyl phthalate (MiBP); mono-n-butyl phthalate (MnBP/MBP); mono-benzyl phthalate (MBzP); mono-(2-ethylhexyl) phthalate (MEHP); mono-(2-ethyl-5-hydroxy-hexyl) phthalate (MEHHP); mono-(2-ethyl-5-oxo-hexyl) phthalate (MEOHP); mono-(2-ethyl-5-carboxylplentyl phthalate (MECPP); mono-cyclo-hexyl phthalate (MCHP); mono-(3-carboxypropyl) phthalate (MCPP); mono-n-octyl phthalate (MnOP/MOP); mono-isononyl phthalate (MiNP). In addition to the individual phthalate metabolites, total phthalate concentration (calculated as the sum of the individual metabolite mass concentrations) was also derived.

### Laboratory methods for phthalate analysis

One mL of thawed human urine was poured into a glass culture tube, buffered with ammonium acetate (250 µL, 1 M, pH = 6.5) and E. coli β-glucuronidase enzyme (5 µL, 200 U/mL) with a mixture of isotope phthalate monoester standards (10 µL). The samples were incubated in a 37 °C water bath for 90 min and then diluted with 1 mL of pH 2 phosphate buffer (0.14 M NaH2PO4 in 1.2% H3PO4) prior to loading onto solid-phase extraction (SPE) cartridges. Commercially available 60 mg/3 mL styrene-divinylbenzene methacrylate copolymer SPE cartridges (NEXUS ABS ELUT, Agilent, CA, USA) were conditioned with 1 mL of acetyl acetate, 1 mL of acetonitrile and 1 mL of pH 2 phosphate buffer. The 2 mL mixture of urine sample phosphate buffer was loaded onto the SPE cartridge at a rate of 1 mL/min. The column was rinsed with 2 mL of 0.1 M formic acid, and then 1 mL of water. The SPE cartridges were dried by passing air through the column for 0.5 min. The phthalate analytes were then eluted with 1 mL of acetonitrile followed by 1 mL of ethyl acetate at 1 mL/min. The eluates were combined and evaporated to dryness under a steady stream of nitrogen gas in a nitrogen evaporator (Organomation Associates Inc., MA, USA) at 50 °C. The residue was resuspended in 200 µL of water for LC/MS/MS analysis.

Isotope labelled internal standards were purchased from Cambridge Isotope Laboratories, Inc (Andover, MA, USA). Ammonium acetate (> 98%) and formic acid were purchased from Sigma Aldrich (St. Louis, MO, USA). Ethyl acetate, acetonitrile, and water (LC/MS grade) were purchased from Merck (Darmstadt, Germany). Quality control procedures included the use of isotope-labelled internal standards, reagent blanks, and calibration checks to ensure accuracy and precision across analytical runs. Chromatographic analysis followed validated methods described by Silva et al. [28]., involving enzymatic deconjugation, solid-phase extraction, high-performance liquid chromatography, and tandem mass spectrometry [28]. 

Urinary phthalate levels were normalised for dilution by specific gravity [29]. The correction formula was Pc = P × [(1.024 − 1)/(SG-1)], where Pc is the specific gravity-corrected phthalate metabolite concentration (ng/mL), P is the experimental phthalate metabolite concentration (ng/mL), and SG is the specific gravity of the urine sample. Specific gravity was measured using a handheld refractometer (RHC-200ATC, Adelab Scientific, Australia), which was calibrated with deionized water before each measurement.

### Sample size and statistical analysis

A sample size of 605 women provides around 80% power to detect an overall effect of phthalate concentration quartile on the mean duration of gestation, with a difference in means of 6 days between the lowest and highest quartiles and an estimated SD of 17.8 days [30], based on a one-way ANOVA (α = 0.05). Phthalate concentrations were summarised descriptively by medians, interquartile ranges (IQR) and geometric means, and categorised into quartiles or log-transformed for analysis due to their skewed distribution. MnOP/MOP and MiNP were dichotomised into presence or absence for analysis due to large numbers of samples where values fell below the limit of detection. To examine the association between phthalate concentrations and gestational age, linear regression models were used. Results are presented as the change in mean gestational age associated with each phthalate quartile compared to the lowest quartile, along with the p-value for the global test of the effect of each phthalate, or with the phthalate detected versus not detected. Both unadjusted effects and effects adjusted for potential confounders (maternal age, BMI, income, socioeconomic status (measured by the Socio-Economic Indexes for Areas Index of Relative Socio-economic Advantage SEIFA IRSAD), secondary school completion, smoking and alcohol consumption, and randomised treatment group) were estimated. Given the relatively small number of preterm birth cases, exploratory analyses were conducted to examine the unadjusted association between phthalate concentrations and preterm birth (< 37 weeks’ gestation), expressed as relative risks (RRs). These results should be interpreted with caution due to the lack of control for confounding factors. Associations between maternal characteristics and log-transformed or dichotomised phthalate concentrations were examined using univariate linear and log binomial regression models, respectively. Results are presented as geometric mean ratios (GMRs) or RRs associated with a 1 unit increase in continuous characteristics or compared to the reference category for categorical characteristics. Statistical significance was assessed at the 0.05 level with no adjustment for multiple comparisons. All analyses were performed using R Statistical Software (v4.2.1, R Core Team 2022).

## Results

**Fig. 1:**
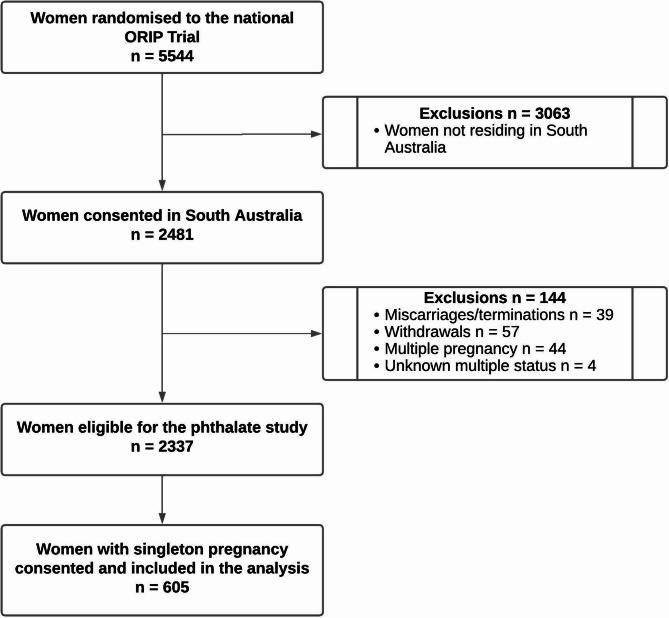
Participant flow for phthalate analysis nested within the ORIP Trial. ORIP, Omega-3 to reduce the incidence of preterm birth

Between November 2014 and October 2016, a total of 618 women from two South Australian Centres consented to this nested phthalate sub-study. Analysis was restricted to singleton pregnancies and excluded women who had withdrawn from the ORIP Trial, resulting in a final sample size of 605 women (24% of the 2481 women enrolled in the ORIP Trial from South Australia), Fig. 1.

### Characteristics of participants

The median age of participants was 30 years. The majority of women were Caucasian (80.8%), with 79.8% having completed secondary school and 83.0% reporting further study. Annual total household income varied with 37% of women reporting household earnings of greater than $105,000AUD per year, while 9% reported income of $40,000 or less. The mean Socio-Economic Indexes for Areas (SEIFA), Index of Relative Socio-Economic Advantage and Disadvantage (IRSAD) score was 990.4, with a standard deviation of 60.9, indicating moderate variability in the socio-economic advantage and disadvantage across the sample. Mean (SD) body mass index at trial entry was 26.7 (5.9) and alcohol consumption (2.5%) and smoking (5.6%) were uncommon among participants at study entry. Slightly more than half of the women (53%) were multiparous and 49% of infants were male, Table 1.

**Table 1:** Baseline demographic characteristics

Characteristic	Total (*n* = 605)
Maternal age: Median (IQR)	30.0 (27.0–34.0)
Caucasian: N(%)	489 (80.8)
Primiparous: N(%)	323 (53.4)
Mother completed secondary school: N(%)	483 (79.8)
Mother completed further study: N(%)	502 (83.0)
Highest qualification completed: N(%)	
No further study	103 (17.0)
Certificate	133 (22.0)
Degree	226 (37.4)
Diploma	67 (11.1)
Higher Degree	74 (12.2)
Other	2 (0.3)
Annual total household income: N(%)	
>$105,001	224 (37.0)
$70,001 - $105,000	141 (23.3)
$40,001 - $70,000	103 (17.0)
$40,000 or less	54 (8.9)
Prefers not to disclose	83 (13.7)
Maternal weight (kg) at study entry: Mean (SD)	72.8 (17.1)
Maternal height (cm) at study entry: Mean (SD)	165.0 (6.7)
BMI: Mean (SD)	26.7 (5.9)
Smoking at study entry: N(%)	34 (5.6)
Smoked leading up to pregnancy: N(%)	85 (14.0)
SEIFA - IRSAD: Mean (SD)	990.4 (60.9)
Alcohol use at study entry: N(%)	15 (2.5)
Male infant: N(%)	299 (49.4)

*BMI* Body mass index, *IRSAD* Index of Relative Socio-economic Advantage and Disadvantage, *IQR* Interquartile range, *SD* Standard deviation, SEIFA Socio-Economic Indexes for Areas

### Urinary phthalate metabolite concentrations

Eleven of the 13 phthalate metabolites analysed were detected in > 99% of maternal urine samples, including MBP, MiBP, MEP, MBzP, MMP, MEHP, MEHHP, MEOHP, MECPP, MCHP, MCPP. There was substantial variability in metabolite concentrations between participants, as indicated by the wide interquartile ranges (IQR), Table 2. Phthalate metabolites with the highest geometric means were MEP 56.7 ng/mL, MiBP 36.0 ng/mL and MBP 27.4 ng/mL, all low molecular weight phthalates commonly found in personal care products. The sum of total phthalate metabolites varied widely among participants, with concentrations ranging from 29.4 ng/mL to 4,839 ng/mL. The median concentration of total phthalates was 224.9 ng/mL, with an IQR of 139.3 to 366.9 ng/mL, indicating considerable variability in phthalate exposure within the population.

**Table 2:** Distribution of specific gravity adjusted urinary levels of phthalate metabolites: quartiles and summary statistics (ng/mL), *n* = 605

Phthalate Metabolite	Detection rate (%> LOD)	Quartiles					Geometric mean
		Minimum	Q1	Median	Q3	Maximum	
MMP	99.7	0.01	3.74	6.55	11.76	2,421	6.36
MEP	100.0	3.46	24.4	49.5	120.4	2,321	56.7
MiBP	99.8	3.02	20.1	35.9	59.6	588.7	34.0
MnBP/MBP	99.8	0.05	16.4	27.4	44.0	527.1	27.4
MBzP	100.0	0.05	2.46	4.82	9.10	700.2	4.87
MEHP	99.8	0.01	1.46	3.38	6.64	150.1	2.84
MEHHP	99.7	0.01	3.69	7.92	14.9	398.2	6.88
MEOHP	99.5	0.01	5.23	9.35	16.7	410.6	8.92
MECPP	99.7	0.01	8.35	15.6	27.8	266.4	14.5
MCHP	100.0	0.12	0.23	0.30	0.42	2.76	0.32
MCPP	99.8	0.01	1.52	2.68	4.61	208.3	2.87
MnOP/MOP	5.3	N/A	N/A	N/A	N/A	14.75	N/A
MiNP	13.3	N/A	N/A	N/A	N/A	41.2	N/A
Total Phthalates	100.0	29.4	139.3	224.9	366.9	4,839	234.0

*LOD* Limit of detection, Mono-methyl phthalate (*MMP*), Mono-ethyl phthalate (*MEP*), Mono-isobutyl phthalate (*MiBP*), Mono-n-butyl phthalate (*MnBP/MBP*), Monobenzyl phthalate (*MBzP*), Mono-(2-ethylhexyl) phthalate (*MEHP*), Mono-(2-ethyl-5-hydroxy-hexyl) phthalate (*MEHHP*), Mono-(2-ethyl-5-oxo-hexyl) phthalate (*MEOHP*), Mono-(2-ethyl-5-carboxylplentyl phthalate (*MECPP*), Mono-cyclo-hexyl phthalate (*MCHP*), Mono-(3-carboxypropyl) phthalate (*MCPP*), Mono-n-octyl phthalate (*MnOP*/*MOP*), Mono-isononyl phthalate (*MiNP*)

### Relationship between urinary phthalate metabolite concentrations and gestational duration

In the unadjusted and adjusted linear regression analyses, there was little evidence of an association between phthalate concentrations and gestational age. There was some suggestion of an association for MEP, where the second quartile was associated with a 3.87-day increase in gestational age in the adjusted analysis (95% CI 0.65–7.10, *P* = 0.02). However, the higher quartiles, Q3 (adjusted mean difference: 0.99 days, *P* = 0.55) and Q4 (adjusted mean difference: 1.74 days, *P* = 0.29), did not significantly differ from the reference group, and the global *P*-value was not significant (*P* = 0.11). Other individual phthalates showed no significant associations with gestational age in either the unadjusted or adjusted models. Overall, there was no consistent pattern of association between the sum of total phthalate concentrations and gestational age, as indicated by non-significant global *P*-values for both unadjusted (*P* = 0.89) and adjusted models (*P* = 0.85), with the mean concentration ranging between 272.8 and 274.2 across quartiles (SD 11.8–15.7), Table 3.

**Table 3:** Effect of phthalates on gestational age

Label		Gestational age mean (SD) days	Unadjusted Effect	*P*	Adjusted Effect^‡^	*P*
MMP	Q1 (< 3.74)	274.1 (15.4)	Reference	0.30^#^	Reference	0.42^#^
	Q2 (≥ 3.74 to < 6.55)	271.8 (13.4)	−2.38 (−5.60, 0.84)	0.15	−1.82 (−5.09, 1.45)	0.28
	Q3 (≥ 6.55 to < 11.76)	274.7 (14.1)	0.58 (−2.63, 3.80)	0.72	0.92 (−2.33, 4.18)	0.58
	Q4 (≥ 11.76)	273.5 (14.1)	−0.68 (−3.89, 2.53)	0.68	−0.26 (−3.53, 3.01)	0.88
MEP	Q1 (< 24.41)	272.3 (17.4)	Reference	0.12^#^	Reference	0.11^#^
	Q2 (≥ 24.41 to < 49.54)	275.9 (9.8)	3.63 (0.42, 6.84)	0.03	3.87 (0.65, 7.10)	0.02
	Q3 (≥ 49.54 to < 120.37)	273.0 (15.1)	0.70 (−2.52, 3.91)	0.67	0.99 (−2.25, 4.24)	0.55
	Q4 (≥ 120.37)	272.9 (13.5)	0.67 (−2.54, 3.87)	0.68	1.74 (−1.50, 4.97)	0.29
MEHHP	Q1 (< 3.69)	273.2 (14.2)	Reference	0.43^#^	Reference	0.45^#^
	Q2 (≥ 3.69 to < 7.92)	272.1 (17.8)	−1.14 (−4.36, 2.08)	0.49	−0.80 (−4.04, 2.44)	0.63
	Q3 (≥ 7.92 to < 14.93)	274.5 (12.5)	1.25 (−1.97, 4.46)	0.45	1.80 (−1.45, 5.05)	0.28
	Q4 (≥ 14.93)	274.4 (11.8)	1.14 (−2.07, 4.36)	0.49	0.59 (−2.64, 3.82)	0.72
MiBP	Q1 (< 20.05)	275.1 (13.0)	Reference	0.35^#^	Reference	0.46^#^
	Q2 (≥ 20.05 to < 35.88)	272.8 (17.4)	−2.26 (−5.48, 0.95)	0.17	−1.96 (−5.24, 1.31)	0.24
	Q3 (≥ 35.88 to < 59.55)	273.9 (11.1)	−1.13 (−4.34, 2.09)	0.49	−1.15 (−4.43, 2.13)	0.49
	Q4 (≥ 59.55)	272.3 (14.7)	−2.71 (−5.92, 0.50)	0.10	−2.56 (−5.87, 0.75)	0.13
MnBP/MBP	Q1 (< 16.4)	275.3 (9.3)	Reference	0.26^#^	Reference	0.33^#^
	Q2 (≥ 16.4 to < 27.38)	272.2 (18.8)	−3.05 (−6.26, 0.17)	0.06	−2.62 (−5.90, 0.66)	0.12
	Q3 (≥ 27.38 to < 44.03)	272.8 (13.8)	−2.50 (−5.72, 0.71)	0.13	−2.56 (−5.82, 0.70)	0.12
	Q4 (≥ 44.03)	273.8 (13.6)	−1.52 (−4.73, 1.70)	0.36	−1.10 (−4.40, 2.20)	0.51
MEOHP	Q1 (< 5.23)	272.5 (16.6)	Reference	0.60^#^	Reference	0.75^#^
	Q2 (≥ 5.23 to < 9.35)	274.0 (13.7)	1.53 (−1.69, 4.75)	0.35	1.50 (−1.73, 4.72)	0.36
	Q3 (≥ 9.35 to < 16.67)	273.1 (14.6)	0.57 (−2.66, 3.80)	0.73	0.51 (−2.76, 3.77)	0.76
	Q4 (≥ 16.67)	274.5 (11.9)	2.03 (−1.18, 5.24)	0.22	1.49 (−1.73, 4.72)	0.37
MBzP	Q1 (< 2.46)	274.6 (9.1)	Reference	0.50^#^	Reference	0.49^#^
	Q2 (≥ 2.46 to < 4.82)	272.3 (17.3)	−2.32 (−5.54, 0.90)	0.16	−2.35 (−5.59, 0.90)	0.16
	Q3 (≥ 4.82 to < 9.1)	273.1 (16.0)	−1.54 (−4.76, 1.68)	0.35	−1.30 (−4.57, 1.97)	0.44
	Q4 (≥ 9.1)	274.1 (13.4)	−0.54 (−3.75, 2.68)	0.74	−0.38 (−3.69, 2.92)	0.82
MCHP	Q1 (< 0.23)	272.0 (14.3)	Reference	0.46^#^	Reference	0.37^#^
	Q2 (≥ 0.23 to < 0.3)	273.7 (16.7)	1.71 (−1.62, 5.05)	0.31	1.56 (−1.84, 4.96)	0.37
	Q3 (≥ 0.3 to < 0.42)	274.6 (14.6)	2.68 (−0.61, 5.98)	0.11	3.03 (−0.33, 6.39)	0.08
	Q4 (≥ 0.42)	273.4 (10.6)	1.47 (−1.90, 4.84)	0.39	1.46 (−1.98, 4.89)	0.41
MCPP	Q1 (< 1.52)	272.4 (16.8)	Reference	0.71^#^	Reference	0.62^#^
	Q2 (≥ 1.52 to < 2.68)	273.5 (13.9)	1.08 (−2.15, 4.31)	0.51	1.21 (−2.04, 4.46)	0.47
	Q3 (≥ 2.68 to < 4.61)	273.8 (13.8)	1.33 (−1.90, 4.56)	0.42	1.08 (−2.17, 4.34)	0.51
	Q4 (≥ 4.61)	274.3 (12.4)	1.89 (−1.33, 5.10)	0.25	2.20 (−1.05, 5.46)	0.19
MECPP	Q1 (< 8.35)	273.2 (16.9)	Reference	0.89^#^	Reference	0.85^#^
	Q2 (≥ 8.35 to < 15.59)	274.0 (13.1)	0.79 (−2.44, 4.01)	0.63	1.17 (−2.09, 4.43)	0.48
	Q3 (≥ 15.59 to < 27.8)	274.0 (12.5)	0.75 (−2.47, 3.98)	0.65	0.73 (−2.52, 3.97)	0.66
	Q4 (≥ 27.8)	272.9 (14.3)	−0.26 (−3.48, 2.96)	0.88	−0.08 (−3.33, 3.16)	0.96
MEHP	Q1 (< 1.46)	272.5 (17.5)	Reference	0.38^#^	Reference	0.67^#^
	Q2 (≥ 1.46 to < 3.38)	272.8 (14.6)	0.33 (−2.89, 3.55)	0.84	0.19 (−3.07, 3.45)	0.91
	Q3 (≥ 3.38 to < 6.64)	273.6 (12.0)	1.09 (−2.13, 4.31)	0.51	0.47 (−2.79, 3.74)	0.78
	Q4 (≥ 6.64)	275.1 (12.2)	2.63 (−0.58, 5.84)	0.11	1.86 (−1.40, 5.12)	0.26
MnOP/MOP	Not detected	273.6 (14.0)	Reference		Reference	
	Detected	272.0 (18.8)	−1.61 (−6.69, 3.47)	0.53	−0.92 (−6.00, 4.16)	0.72
MiNP	Not detected	273.4 (14.7)	Reference		Reference	
	Detected	274.8 (8.9)	1.41 (−2.61, 5.43)	0.49	1.44 (−2.63, 5.50)	0.49
Total Phthalates	Q1 (< 139.3)	273.6 (15.0)	Reference	0.89^#^	Reference	0.85^#^
	Q2 (≥ 139.3 to < 224.93)	274.2 (14.4)	0.56 (−2.66, 3.79)	0.73	1.35 (−1.91, 4.61)	0.42
	Q3 (≥ 224.93 to < 366.85)	273.5 (15.7)	−0.09 (−3.31, 3.14)	0.96	0.28 (−2.99, 3.56)	0.87
	Q4 (≥ 366.85)	272.8 (11.8)	−0.75 (−3.97, 2.47)	0.65	0.16 (−3.09, 3.41)	0.92

*Q1-4 Q*uartiles 1–4^‡^ Adjusted for maternal age, *BMI* Income, *SES *(*SEIFA IRSAD*) Education (completed secondary school), Smoking and alcohol consumption, and treatment group

^#^Global test for overall effect of phthalate quartiles

### Relationship between urinary phthalate metabolite concentrations and preterm birth

A total of 47 infants in this study were born preterm (< 37 weeks’ gestation), representing 7.7% of the study population, slightly less than the national average of 8.5 to 8.7% during this period. The exploratory, unadjusted log-binomial regression analyses for preterm birth (< 37 weeks’ gestation) suggested no significant associations with maternal phthalate concentrations, (Supplementary Table 2, Additional File 1).

### Relationship between maternal characteristics and urinary phthalate metabolite concentrations

Since this analysis aimed to assess the relationship between phthalate concentrations and gestational length, selected maternal characteristics were included as potential confounders in the analysis. Results from univariate analyses estimating changes in mean log-transformed phthalate metabolite levels for each maternal characteristic separately, suggested that individual phthalate concentrations were associated with certain maternal characteristics, (Supplementary Table 3, Additional File 1).

Smoking leading up to pregnancy was associated with increased concentrations of several phthalate metabolites including MMP (geometric mean ratio (GMR) 1.37, 95%CI 1.04 to 1.79; *P* = 0.03), MEP (GMR 1.35, 95%CI 1.02 to 1.77, *P* = 0.04) and MBzP (GMR 1.54, 95%CI 1.19 to 2.01, *P* = 0.001). Higher body mass index (BMI) was linked to increases in MEP, (GMR 1.02, 95% CI 1.00 to 1.03, *P* = 0.04) whereas MEHP concentrations were associated with reductions in BMI (GMR 0.97, 95% CI 0.96 to 0.99, *P* = 0.008).

Household income was also associated with phthalate metabolite concentrations, with lower-income women exhibiting higher concentrations of several phthalates. A progressive decrease in MiBP, MBP, and MBzP concentrations was observed with each rise in income bracket (global P-values < 0.0001, < 0.0001, and 0.001, respectively). These three metabolites are all found in personal care products. Similarly, women with higher educational attainment had lower concentrations of MBzP and MOP.

Given that this study was conducted within the framework of a randomised controlled trial of omega-3 supplementation, participation in the intervention arm was also examined as a maternal characteristic. Women randomized to receive 900 mg of omega-3 daily (800 mg DHA/100 mg EPA) had lower concentrations of some phthalate metabolites, including MMP (GMR 0.81, 95% CI 0.67–0.99, *P* = 0.03) and MCPP (GMR 0.77, 95% CI 0.65–0.91, *P* = 0.003), compared to those in the control arm.

## Discussion

In this prospective cohort study, nested within a large randomised controlled trial, we examined the relationship between maternal phthalate exposure, gestational length, and preterm birth, finding no significant associations across thirteen phthalate metabolites. While several studies have reported associations between higher phthalate concentrations and an increased risk of preterm birth or shortened gestational length, others, including ours, have found no clear relationship.

The detection of eleven out of thirteen urinary phthalate metabolites in over 99% of samples highlights widespread exposure among pregnant women in South Australia. MEP, a metabolite of diethyl phthalate (DEP), had the highest mean concentration (49.5 µg/L), aligning with research identifying MEP as a predominant metabolite in pregnant populations [14, 31–33]. Despite this ubiquitous exposure, our findings contrast with several studies, particularly from the United States, that have reported associations between higher phthalate concentrations and reduced gestational age or increased risk of preterm birth.

For example, Ferguson et al. found that higher urinary concentrations of MEHP, MECPP, and the molar sum of di(2-ethylhexyl) phthalate (DEHP) metabolites were significantly associated with an increased risk of spontaneous preterm birth in a nested case-control study [34]. Similarly, a cohort study in New York City reported that higher MEHP levels were associated with reduced gestational age after adjusting for key confounders [35]. A pooled analysis of 16 U.S. cohorts (*n* = 6,045) found a 12–16% increase in preterm birth odds per interquartile range increase in MiBP, MBP, MCPP, and MECPP [13]. Similar associations have been reported in Poland [36], China [37], Italy [38], Mexico [39], and Denmark [40].

However, not all studies report adverse associations, and some have even observed opposing trends. Gao et al. found non-linear associations in a cohort of 3,266 women in China, suggesting that certain phthalates may be linked to both preterm and post-term birth [41]. Adibi et al. reported a 1.1–1.3-day increase in gestational length per log-unit increase in urinary DEHP metabolites (MEHP, MEOHP) in a U.S. cohort [42]. Other studies from the U.S. and Spain have either found no association [43] or positive associations with increased gestational length [42, 44]. Our study also observed a non-linear association, with a 3.87-day increase in gestational age in the second quartile of MEP exposure (95% CI 0.65–7.10, *P* = 0.02). However, higher quartiles (Q3 and Q4) did not show significant differences from the reference group, and the global P-value was not significant (*P* = 0.11).

Variability in findings between studies likely stems from differences in study design, confounder adjustment, and exposure levels. International variations in product regulations, manufacturing practices, consumer habits, and regulatory enforcement may further contribute to disparities in phthalate exposure.

Our Australian cohort had lower urinary phthalate concentrations than some U.S. studies, particularly for DEHP metabolites, which may have influenced our ability to detect associations. For example, compared to Ferguson et al. [35] median levels in our study vs. their U.S. cohort were: MEHP 3.4 ng/mL vs. 10.5 ng/mL, MEOHP 9.4 ng/mL vs. 16.9 ng/mL, MECPP 15.6 ng/mL vs. 41.3 ng/mL, and MEHHP 7.9 ng/mL vs. 31.9 ng/mL. While these differences suggest that a threshold level of exposure may influence detectability of effects, findings from other studies complicate this interpretation. For instance, Polanska et al. [36] reported inverse associations with gestational age despite lower concentrations than ours, and Abidi et al. [42] found positive associations with DEHP metabolites at levels similar to or slightly higher than those observed here. Together, these mixed findings point to the complexity of phthalate–pregnancy outcome relationships and suggest that factors beyond exposure level, including population characteristics, timing of exposure measurement, and study design, may also contribute to inconsistencies in observed associations.

Within our study, phthalate metabolite concentrations varied significantly between individuals, reflecting differences in environmental exposures. Factors such as product usage, dietary habits, and lifestyle choices likely contribute to these variations. Our analysis identified several maternal characteristics associated with phthalate levels, consistent with previous studies [45, 46].

Smoking prior to pregnancy was linked to increased levels of several phthalate metabolites, specifically MMP, MEP, and MBzP (RR 1.37, 1.35, and 1.54 respectively), suggesting that tobacco exposure may elevate phthalate levels. This could be due to the presence of phthalates in tobacco smoke or cigarette packaging. Smoking is also more prevalent among women with lower socioeconomic status, which may partly explain the higher phthalate concentrations observed in this group. Higher maternal BMI was linked to increased MEP levels (RR 1.02) and decreased MEHP levels (RR 0.97). This could suggest that body composition influences phthalate metabolism, or that individuals with higher BMI may have different dietary patterns, including greater consumption of processed and packaged foods, a major source of phthalate exposure. Socioeconomic disparities were evident, with women with lower-income women exhibiting higher concentrations of MiBP, MBP, and MBzP (global P-values < 0.0001, < 0.0001, and 0.001 respectively), while women with higher educational attainment had lower MBzP and MOP levels. Although our cohort predominantly identified as Caucasian, limiting the ability to examine ethnic differences, these findings align with broader evidence that phthalate exposure is not equally distributed across populations, with socially disadvantaged women potentially at higher risk of exposure [47–49]. Understanding these influences is crucial for identifying at-risk populations and promoting strategies to minimise exposure.

Women randomised to the intervention arm of the ORIP Trial, receiving daily 900 mg of omega-3 long-chain polyunsaturated fatty acids (LCPUFA), had lower levels of MMP and MCPP [24]. Omega-3 LCPUFA are well-recognised anti-inflammatory agents with the potential to influence a range of biological processes [50, 51]. Although the mechanistic role of omega-3 LCPUFA in modifying phthalate metabolism remains unclear, there are plausible biological pathways. Omega-3 LCPUFA may mitigate phthalate-mediated effects by modulating cyclooxygenase-2 (COX-2) expression and reducing the release of pro-inflammatory prostaglandins [52]. Additionally, they may counteract oxidative stress and inflammatory responses associated with phthalate exposure [53]. Further research is needed to explore whether omega-3 LCPUFA could mitigate the effects of phthalate exposure, particularly in high-risk populations.

A key strength of this study is its nested design within a large, rigorously conducted randomised controlled trial. By leveraging this trial framework, our study benefited from rigorous control of sociodemographic and lifestyle factors, potentially leading to more reliable effect estimates. Additionally, urine samples were collected between 22 and 26 weeks’ gestation, an optimal window for assessing potential associations with preterm birth [54]. Although multiple urine samples throughout pregnancy would have been ideal, evidence suggests that single spot urine samples adequately reflect phthalate exposure, given its environmental persistence and consistent daily exposure patterns [55, 56].

A limitation is the small number of preterm birth cases, reducing the statistical power to detect associations and limiting our ability to adjust for important potential confounders, such as maternal age and BMI. Adjusted effects of phthalates on preterm birth should therefore be explored in future larger studies. However, by analysing gestational length as a continuous variable, we were able to capture subtle variations in pregnancy duration and control for important confounders, thus providing valuable insights into potential effects associated with phthalate exposure.

## Conclusion

Despite widespread phthalate exposure among pregnant women in South Australia, our findings suggest that exposure levels in this population are lower than those linked to shortened pregnancy duration in previous studies. However, phthalates remain pervasive in personal care products, food packaging, and household items, and their potential health effects remain a global concern. Given the breadth of adverse maternal and infant outcomes reported in the literature [20], minimising exposure during pregnancy is likely to help protect long-term health of mothers and babies.

With the increasing use of substitute plasticisers and phthalate replacements, ongoing monitoring is essential to evaluate the safety and potential effects of these alternative compounds on pregnancy and fetal development. As consumer products evolve and regulatory landscapes shift, sustained surveillance of maternal exposure will be critical in shaping evidence-based public health policies and helping to prevent potential adverse effects of phthalate exposure on maternal and child health.

## Supplementary Information

Supplementary Material 1.

## Data Availability

De-identified data may be shared upon reasonable request. Proposals for data access must be scientifically and methodologically sound and will be reviewed and approved by the ORIP Trial Steering Committee and the Women’s and Children’s Health Network Human Research Ethics Committee.

## Abbreviations

BMI: Body mass index
CI: Confidence interval
COX: 2–Cyclooxygenase–2
DEHP: Di(2–ethylhexyl) phthalate
DEP: Diethyl phthalate
DHA: Docosahexaenoic acid
EPA: Eicosapentaenoic acid
GMR: Geometric mean ratio
IRSAD: Index of relative socio–economic advantage and disadvantage
IQR: Interquartile range
LC/MS/MS: Liquid chromatography–tandem mass spectrometry
LOD: Limit of detection
LCPUFA: Long–chain polyunsaturated fatty acids
MECPP: Mono–(2–ethyl–5–carboxypentyl) phthalate
MEHP: Mono–(2–ethylhexyl) phthalate
MEHHP: Mono–(2–ethyl–5–hydroxy–hexyl) phthalate
MEOHP: Mono–(2–ethyl–5–oxo–hexyl) phthalate
MCHP: Mono–cyclo–hexyl phthalate
MCPP: Mono–(3–carboxypropyl) phthalate
MBP/MnBP: Mono–n–butyl phthalate
MBzP: Mono–benzyl phthalate
MEP: Mono–ethyl phthalate
MiBP: Mono–isobutyl phthalate
MiNP: Mono–isononyl phthalate
MnOP/MOP: Mono–n–octyl phthalate
MMP: Mono–methyl phthalate
NHMRC: National health and medical research council
ORIP: Omega–3 to reduce the incidence of prematurity trial
PVC: Polyvinyl chloride
RR: Relative risk
SAHMRI: South Australian health and medical research institute
SD: Standard deviation
SEIFA: Socio–economic indexes for areas
SG: Specific gravity
SPE: Solid phase extraction
