# Supplementary material for: Maternal phthalate exposure, gestational length, and preterm birth risk: a prospective cohort study nested within a randomised trial
Source: BMC Pregnancy Childbirth. 2025 Aug 8;25:830. doi: 10.1186/s12884-025-07980-8 (PMC12335031; doi:10.1186/s12884-025-07980-8)
Supplement: Supplementary file 1 — Supplementary Material 1. [file 12884_2025_7980_MOESM1_ESM.docx]

**Supplementary Table 1. Parent compounds their metabolites and common usage**

| **Parent Compound** | **Phthalate Metabolite** | **Common Usage** |
| --- | --- | --- |
| Low Molecular Weight | | |
| Dimethyl phthalate (DMP) | Mono-methyl phthalate (MMP) | Personal care products (e.g. fragrances, cosmetics), insect repellents, solvents, lacquers, paints, plastics and rubbers |
| Diethyl phthalate (DEP) | Mono-ethyl phthalate (MEP) | Personal care products (e.g. fragrances, cosmetics, soap), food packaging, coatings (e.g. pharmaceuticals), toys, dyes, insecticides, industrial solvents |
| Di-n-butyl phthalate (DnBP) | Mono-isobutyl phthalate (MiBP), Mono-n-butyl phthalate (MnBP/MBP) | Personal care products (e.g. nail polish, cosmetics), adhesives, industrial solvent, coatings (e.g. pharmaceuticals), lacquers, varnishes printing ink |
| Butyl benzyl phthalate (BBzP) | Mono-benzyl phthalate (MBzP) | Personal care products, toys, food packaging, vinyl flooring, adhesives and sealants, industrial solvents |
| High Molecular Weight | | |
| Di(2-ethylhexyl) phthalate (DEHP) | Mono-(2-ethylhexyl) phthalate (MEHP), Mono-(2-ethyl-5-hydroxy-hexyl) phthalate (MEHHP), Mono-(2-ethyl-5-oxo-hexyl) phthalate (MEOHP), Mono-(2-ethyl-5-carboxypentyl phthalate) (MECPP) | Soft plastics including tubing, household products, toys, furniture upholstery, food containers, food packaging, blood storage bags and medical devices |
| Di-cyclo-hexyl phthalate (DCHP) | Mono-cyclo-hexyl phthalate (MCHP) | Stabiliser in rubber, polymers, food wrappers, pharmaceutical labels |
| Di-n-octyl phthalate (DnOP/DOP) | Mono-(3-carboxypropyl) phthalate (MCPP), Mono-n-octyl phthalate (MnOP/MOP) | Plasticiser in automotive and industrial hose, conveyer belts, insulation materials, polyurethane surface coatings, floor finishes and adhesives. Toys, play and exercise balls |
| Di-iso-nonyl phthalate (DINP) | Mono-isononyl phthalate (MiNP) | Plasticiser for polyvinyl chloride, adhesives, laminations, resins, surfactants, screen printing inks, toys |

**Supplementary Table 2. Unadjusted Effect of Phthalates on Preterm Birth (<37 weeks’ gestation)**

| **Label** |  | **Preterm**  **N(%)** | **Unadjusted**  **RR (95% CI)** | **P** |
| --- | --- | --- | --- | --- |
| MMP | Q1 (<3.74) | 10 (6.6) | Reference | 0.16^#^ |
|  | Q2 (≥3.74 to <6.55) | 17 (11.3) | 1.71 (0.81, 3.61) | 0.16 |
|  | Q3 (≥6.55 to <11.76) | 7 (4.6) | 0.70 (0.27, 1.78) | 0.45 |
|  | Q4 (≥11.76) | 13 (8.6) | 1.29 (0.58, 2.85) | 0.53 |
| MEP | Q1 (<24.41) | 15 (9.9) | Reference | 0.05^#^ |
|  | Q2 (≥24.41 to <49.54) | 5 (3.3) | 0.33 (0.12, 0.89) | 0.03 |
|  | Q3 (≥49.54 to <120.37) | 11 (7.3) | 0.73 (0.35, 1.54) | 0.41 |
|  | Q4 (≥120.37) | 16 (10.5) | 1.06 (0.54, 2.07) | 0.86 |
| MEHHP | Q1 (<3.69) | 11 (7.3) | Reference | 0.47^#^ |
|  | Q2 (≥3.69 to <7.92) | 15 (9.9) | 1.36 (0.65, 2.87) | 0.41 |
|  | Q3 (≥7.92 to <14.93) | 8 (5.3) | 0.73 (0.30, 1.76) | 0.48 |
|  | Q4 (≥14.93) | 13 (8.6) | 1.17 (0.54, 2.54) | 0.68 |
| MiBP | Q1 (<20.05) | 9 (6.0) | Reference | 0.59^#^ |
|  | Q2 (≥20.05 to <35.88) | 14 (9.3) | 1.56 (0.69, 3.48) | 0.28 |
|  | Q3 (≥35.88 to <59.55) | 10 (6.6) | 1.11 (0.46, 2.66) | 0.81 |
|  | Q4 (≥59.55) | 14 (9.2) | 1.55 (0.69, 3.46) | 0.29 |
| MnBP/MBP | Q1 (<16.4) | 8 (5.3) | Reference | 0.47^#^ |
|  | Q2 (≥16.4 to <27.38) | 15 (9.9) | 1.87 (0.82, 4.29) | 0.14 |
|  | Q3 (≥27.38 to <44.03) | 11 (7.3) | 1.37 (0.57, 3.32) | 0.48 |
|  | Q4 (≥44.03) | 13 (8.6) | 1.61 (0.69, 3.78) | 0.27 |
| MEOHP | Q1 (<5.23) | 12 (7.9) | Reference | 0.98^#^ |
|  | Q2 (≥5.23 to <9.35) | 11 (7.3) | 0.92 (0.42, 2.01) | 0.83 |
|  | Q3 (≥9.35 to <16.67) | 11 (7.3) | 0.92 (0.42, 2.03) | 0.84 |
|  | Q4 (≥16.67) | 13 (8.5) | 1.07 (0.50, 2.27) | 0.86 |
| MBzP | Q1 (<2.46) | 10 (6.6) | Reference | 0.33^#^ |
|  | Q2 (≥2.46 to <4.82) | 16 (10.6) | 1.60 (0.75, 3.41) | 0.22 |
|  | Q3 (≥4.82 to <9.1) | 13 (8.6) | 1.30 (0.59, 2.87) | 0.52 |
|  | Q4 (≥9.1) | 8 (5.3) | 0.79 (0.32, 1.96) | 0.62 |
| MCHP | Q1 (<0.23) | 12 (9.5) | Reference | 0.21^#^ |
|  | Q2 (≥0.23 to <0.3) | 11 (6.9) | 0.73 (0.33, 1.59) | 0.42 |
|  | Q3 (≥0.3 to <0.42) | 8 (4.8) | 0.50 (0.21, 1.19) | 0.12 |
|  | Q4 (≥0.42) | 16 (10.5) | 1.11 (0.54, 2.25) | 0.78 |
| MCPP | Q1 (<1.52) | 10 (6.7) | Reference | 0.75^#^ |
|  | Q2 (≥1.52 to <2.68) | 13 (8.6) | 1.29 (0.58, 2.85) | 0.53 |
|  | Q3 (≥2.68 to <4.61) | 14 (9.3) | 1.39 (0.64, 3.03) | 0.41 |
|  | Q4 (≥4.61) | 10 (6.5) | 0.98 (0.42, 2.29) | 0.96 |
| MECPP | Q1 (<8.35) | 14 (9.3) | Reference | 0.25^#^ |
|  | Q2 (≥8.35 to <15.59) | 8 (5.3) | 0.57 (0.25, 1.32) | 0.19 |
|  | Q3 (≥15.59 to <27.8) | 9 (6.0) | 0.64 (0.29, 1.44) | 0.28 |
|  | Q4 (≥27.8) | 16 (10.5) | 1.14 (0.57, 2.24) | 0.71 |
| MEHP | Q1 (<1.46) | 16 (10.6) | Reference | 0.54^#^ |
|  | Q2 (≥1.46 to <3.38) | 10 (6.6) | 0.63 (0.29, 1.33) | 0.22 |
|  | Q3 (≥3.38 to <6.64) | 10 (6.6) | 0.63 (0.29, 1.33) | 0.22 |
|  | Q4 (≥6.64) | 11 (7.2) | 0.68 (0.33, 1.42) | 0.31 |
| MnOP/MOP | Not detected | 44 (7.7) | Reference |  |
|  | Detected | 3 (9.4) | 1.22 (0.40, 3.72) | 0.73 |
| MiNP | Not detected | 46 (8.3) | Reference |  |
|  | Detected | 1 (1.9) | 0.23 (0.03, 1.61) | 0.14 |
| Total Phthalates | Q1 (<139.3) | 10 (6.6) | Reference | 0.86^#^ |
|  | Q2 (≥139.3 to <224.93) | 11 (7.3) | 1.10 (0.48, 2.51) | 0.82 |
|  | Q3 (≥224.93 to <366.85) | 12 (7.9) | 1.20 (0.53, 2.69) | 0.66 |
|  | Q4 (≥366.85) | 14 (9.2) | 1.39 (0.64, 3.03) | 0.41 |

RR, relative risk

^#^ Global test for overall effect of phthalate quartiles

**Supplementary Table 3. Effect of Maternal Characteristics on Phthalate Concentrations**

| **Phthalate** | **Characteristic** | **Category** | **Reference** | **Unadjusted Effect (95% CI)*** | **P** | **P (global)** ^†^ |
| --- | --- | --- | --- | --- | --- | --- |
| MMP | Mother completed secondary school | Yes | No | 1.05 (0.83, 1.33) | 0.68 |  |
|  | Mother completed further studies | Yes | No | 0.96 (0.75, 1.24) | 0.78 |  |
|  | Annual total household income | $40,001 - $70,000 | $40,000 or less | 1.06 (0.71, 1.59) | 0.78 | 0.9 |
|  | Annual total household income | $70,001 - $105,000 | $40,000 or less | 0.95 (0.65, 1.39) | 0.79 |  |
|  | Annual total household income | >$105,001 | $40,000 or less | 0.96 (0.67, 1.38) | 0.83 |  |
|  | BMI |  | Increase of 1 kg/m^2^ | 0.99 (0.97, 1.01) | 0.25 |  |
|  | Currently smoking | Yes | No | 1.27 (0.84, 1.92) | 0.26 |  |
|  | Smoked leading up to pregnancy | Yes | No | 1.37 (1.04, 1.79) | 0.03 |  |
|  | Treatment group | n-3 | Control | 0.81 (0.67, 0.99) | 0.03 |  |
| MEP | Mother completed secondary school | Yes | No | 0.83 (0.65, 1.05) | 0.13 |  |
|  | Mother completed further studies | Yes | No | 0.95 (0.74, 1.23) | 0.70 |  |
|  | Annual total household income | $40,001 - $70,000 | $40,000 or less | 1.11 (0.74, 1.65) | 0.61 | 0.10 |
|  | Annual total household income | $70,001 - $105,000 | $40,000 or less | 0.78 (0.53, 1.14) | 0.20 |  |
|  | Annual total household income | >$105,001 | $40,000 or less | 0.83 (0.58, 1.19) | 0.31 |  |
|  | BMI |  | Increase of 1 kg/m2 | 1.02 (1.00, 1.03) | 0.04 |  |
|  | Currently smoking | Yes | No | 1.21 (0.80, 1.84) | 0.37 |  |
|  | Smoked leading up to pregnancy | Yes | No | 1.35 (1.02, 1.77) | 0.04 |  |
|  | Treatment group | n-3 | Control | 0.86 (0.71, 1.05) | 0.14 |  |
| MEHHP | Mother completed secondary school | Yes | No | 1.06 (0.81, 1.39) | 0.66 |  |
|  | Mother completed further studies | Yes | No | 1.04 (0.78, 1.39) | 0.78 |  |
|  | Annual total household income | $40,001 - $70,000 | $40,000 or less | 0.76 (0.49, 1.20) | 0.24 | 0.6 |
|  | Annual total household income | $70,001 - $105,000 | $40,000 or less | 0.89 (0.58, 1.37) | 0.59 |  |
|  | Annual total household income | >$105,001 | $40,000 or less | 0.81 (0.54, 1.22) | 0.32 |  |
|  | BMI |  | Increase of 1 kg/m2 | 1.00 (0.98, 1.02) | 0.83 |  |
|  | Currently smoking | Yes | No | 1.20 (0.75, 1.93) | 0.44 |  |
|  | Smoked leading up to pregnancy | Yes | No | 1.15 (0.84, 1.58) | 0.37 |  |
|  | Treatment group | n-3 | Control | 0.92 (0.74, 1.14) | 0.45 |  |
| MiBP | Mother completed secondary school | Yes | No | 0.94 (0.80, 1.11) | 0.47 |  |
|  | Mother completed further studies | Yes | No | 0.84 (0.71, 1.00) | 0.05 |  |
|  | Annual total household income | $40,001 - $70,000 | $40,000 or less | 0.88 (0.68, 1.15) | 0.35 | <0.0001 |
|  | Annual total household income | $70,001 - $105,000 | $40,000 or less | 0.72 (0.56, 0.92) | 0.010 |  |
|  | Annual total household income | >$105,001 | $40,000 or less | 0.62 (0.49, 0.78) | <0.0001 |  |
|  | BMI |  | Increase of 1 kg/m2 | 1.01 (1.00, 1.02) | 0.05 |  |
|  | Currently smoking | Yes | No | 1.09 (0.82, 1.45) | 0.54 |  |
|  | Smoked leading up to pregnancy | Yes | No | 1.08 (0.89, 1.30) | 0.44 |  |
|  | Treatment group | n-3 | Control | 0.99 (0.87, 1.12) | 0.84 |  |
| MnBP/MBP | Mother completed secondary school | Yes | No | 0.96 (0.80, 1.14) | 0.61 |  |
|  | Mother completed further studies | Yes | No | 0.96 (0.79, 1.15) | 0.63 |  |
|  | Annual total household income | $40,001 - $70,000 | $40,000 or less | 0.82 (0.61, 1.08) | 0.16 | <0.0001 |
|  | Annual total household income | $70,001 - $105,000 | $40,000 or less | 0.62 (0.47, 0.81) | 0.0006 |  |
|  | Annual total household income | >$105,001 | $40,000 or less | 0.56 (0.44, 0.73) | <0.0001 |  |
|  | BMI |  | Increase of 1 kg/m2 | 1.00 (0.99, 1.01) | 0.73 |  |
|  | Currently smoking | Yes | No | 1.11 (0.82, 1.50) | 0.51 |  |
|  | Smoked leading up to pregnancy | Yes | No | 1.08 (0.89, 1.32) | 0.43 |  |
|  | Treatment group | n-3 | Control | 0.94 (0.82, 1.08) | 0.39 |  |
| MEOHP | Mother completed secondary school | Yes | No | 1.04 (0.83, 1.31) | 0.71 |  |
|  | Mother completed further studies | Yes | No | 0.96 (0.75, 1.22) | 0.72 |  |
|  | Annual total household income | $40,001 - $70,000 | $40,000 or less | 0.83 (0.59, 1.17) | 0.28 | 0.7 |
|  | Annual total household income | $70,001 - $105,000 | $40,000 or less | 0.86 (0.62, 1.19) | 0.37 |  |
|  | Annual total household income | >$105,001 | $40,000 or less | 0.86 (0.63, 1.16) | 0.32 |  |
|  | BMI |  | Increase of 1 kg/m2 | 1.00 (0.99, 1.02) | 0.88 |  |
|  | Currently smoking | Yes | No | 1.28 (0.86, 1.91) | 0.23 |  |
|  | Smoked leading up to pregnancy | Yes | No | 1.15 (0.88, 1.49) | 0.31 |  |
|  | Treatment group | n-3 | Control | 0.92 (0.76, 1.10) | 0.36 |  |
| MBzP | Mother completed secondary school | Yes | No | 0.66 (0.53, 0.83) | 0.0004 |  |
|  | Mother completed further studies | Yes | No | 0.82 (0.64, 1.04) | 0.10 |  |
|  | Annual total household income | $40,001 - $70,000 | $40,000 or less | 0.83 (0.57, 1.21) | 0.33 | 0.001 |
|  | Annual total household income | $70,001 - $105,000 | $40,000 or less | 0.65 (0.46, 0.93) | 0.02 |  |
|  | Annual total household income | >$105,001 | $40,000 or less | 0.56 (0.40, 0.79) | 0.0009 |  |
|  | BMI |  | Increase of 1 kg/m2 | 1.01 (0.99, 1.03) | 0.21 |  |
|  | Currently smoking | Yes | No | 1.76 (1.19, 2.62) | 0.005 |  |
|  | Smoked leading up to pregnancy | Yes | No | 1.54 (1.19, 2.01) | 0.001 |  |
|  | Treatment group | n-3 | Control | 1.02 (0.85, 1.23) | 0.80 |  |
| MCHP | Mother completed secondary school | Yes | No | 1.05 (0.96, 1.15) | 0.25 |  |
|  | Mother completed further studies | Yes | No | 1.07 (0.97, 1.17) | 0.18 |  |
|  | Annual total household income | $40,001 - $70,000 | $40,000 or less | 1.01 (0.88, 1.17) | 0.85 | 0.6 |
|  | Annual total household income | $70,001 - $105,000 | $40,000 or less | 0.95 (0.83, 1.09) | 0.45 |  |
|  | Annual total household income | >$105,001 | $40,000 or less | 0.97 (0.85, 1.10) | 0.59 |  |
|  | BMI |  | Increase of 1 kg/m2 | 0.99 (0.99, 1.00) | 0.09 |  |
|  | Currently smoking | Yes | No | 0.95 (0.81, 1.11) | 0.50 |  |
|  | Smoked leading up to pregnancy | Yes | No | 0.93 (0.84, 1.03) | 0.19 |  |
|  | Treatment group | n-3 | Control | 1.02 (0.95, 1.10) | 0.55 |  |
| MCPP | Mother completed secondary school | Yes | No | 1.09 (0.88, 1.34) | 0.42 |  |
|  | Mother completed further studies | Yes | No | 0.97 (0.77, 1.21) | 0.79 |  |
|  | Annual total household income | $40,001 - $70,000 | $40,000 or less | 0.98 (0.69, 1.40) | 0.91 | 0.7 |
|  | Annual total household income | $70,001 - $105,000 | $40,000 or less | 0.98 (0.70, 1.38) | 0.93 |  |
|  | Annual total household income | >$105,001 | $40,000 or less | 1.10 (0.80, 1.51) | 0.56 |  |
|  | BMI |  | Increase of 1 kg/m2 | 1.00 (0.98, 1.01) | 0.90 |  |
|  | Currently smoking | Yes | No | 0.94 (0.65, 1.35) | 0.73 |  |
|  | Smoked leading up to pregnancy | Yes | No | 1.01 (0.79, 1.29) | 0.95 |  |
|  | Treatment group | n-3 | Control | 0.77 (0.65, 0.91) | 0.003 |  |
| MECPP | Mother completed secondary school | Yes | No | 0.96 (0.77, 1.20) | 0.75 |  |
|  | Mother completed further studies | Yes | No | 0.84 (0.66, 1.06) | 0.14 |  |
|  | Annual total household income | $40,001 - $70,000 | $40,000 or less | 0.84 (0.59, 1.20) | 0.33 | 0.7 |
|  | Annual total household income | $70,001 - $105,000 | $40,000 or less | 0.87 (0.62, 1.22) | 0.41 |  |
|  | Annual total household income | >$105,001 | $40,000 or less | 0.82 (0.60, 1.13) | 0.23 |  |
|  | BMI |  | Increase of 1 kg/m2 | 1.01 (0.99, 1.02) | 0.43 |  |
|  | Currently smoking | Yes | No | 1.20 (0.81, 1.76) | 0.36 |  |
|  | Smoked leading up to pregnancy | Yes | No | 1.13 (0.87, 1.45) | 0.37 |  |
|  | Treatment group | n-3 | Control | 1.03 (0.86, 1.23) | 0.74 |  |
| MEHP | Mother completed secondary school | Yes | No | 0.91 (0.69, 1.19) | 0.49 |  |
|  | Mother completed further studies | Yes | No | 1.00 (0.75, 1.34) | 0.99 |  |
|  | Annual total household income | $40,001 - $70,000 | $40,000 or less | 0.78 (0.50, 1.23) | 0.29 | 0.1 |
|  | Annual total household income | $70,001 - $105,000 | $40,000 or less | 0.88 (0.57, 1.35) | 0.55 |  |
|  | Annual total household income | >$105,001 | $40,000 or less | 0.67 (0.44, 1.01) | 0.05 |  |
|  | BMI |  | Increase of 1 kg/m2 | 0.97 (0.96, 0.99) | 0.008 |  |
|  | Currently smoking | Yes | No | 1.45 (0.90, 2.33) | 0.12 |  |
|  | Smoked leading up to pregnancy | Yes | No | 1.16 (0.85, 1.59) | 0.35 |  |
|  | Treatment group | n-3 | Control | 0.92 (0.74, 1.15) | 0.46 |  |
| MnOP/MOP^#^ | Mother completed secondary school | Yes | No | 0.65 (0.31, 1.36) | 0.25 |  |
|  | Mother completed further studies | Yes | No | 0.39 (0.19, 0.79) | 0.008 |  |
|  | Annual total household income | $40,001 - $70,000 | $40,000 or less | 1.31 (0.26, 6.53) | 0.74 | 0.9 |
|  | Annual total household income | $70,001 - $105,000 | $40,000 or less | 1.53 (0.34, 6.98) | 0.58 |  |
|  | Annual total household income | >$105,001 | $40,000 or less | 1.45 (0.33, 6.27) | 0.62 |  |
|  | BMI |  | Increase of 1 kg/m2 | 0.98 (0.92, 1.04) | 0.56 |  |
|  | Currently smoking | Yes | No | 0.00 (0.00, Inf) | 0.99 |  |
|  | Smoked leading up to pregnancy | Yes | No | 0.41 (0.10, 1.68) | 0.21 |  |
|  | Treatment group | n-3 | Control | 1.21 (0.62, 2.39) | 0.57 |  |
| MiNP^#^ | Mother completed secondary school | Yes | No | 0.98 (0.59, 1.62) | 0.93 |  |
|  | Mother completed further studies | Yes | No | 0.92 (0.55, 1.56) | 0.77 |  |
|  | Annual total household income | $40,001 - $70,000 | $40,000 or less | 1.65 (0.67, 4.05) | 0.27 | 0.6 |
|  | Annual total household income | $70,001 - $105,000 | $40,000 or less | 1.27 (0.52, 3.12) | 0.60 |  |
|  | Annual total household income | >$105,001 | $40,000 or less | 1.19 (0.50, 2.82) | 0.70 |  |
|  | BMI |  | Increase of 1 kg/m2 | 0.99 (0.95, 1.02) | 0.50 |  |
|  | Currently smoking | Yes | No | 1.23 (0.56, 2.72) | 0.61 |  |
|  | Smoked leading up to pregnancy | Yes | No | 1.59 (0.97, 2.59) | 0.06 |  |
|  | Treatment group | n-3 | Control | 0.88 (0.59, 1.33) | 0.56 |  |
| Total Phthalates | Mother completed secondary school | Yes | No | 0.98 (0.84, 1.14) | 0.79 |  |
|  | Mother completed further studies | Yes | No | 0.92 (0.79, 1.08) | 0.32 |  |
|  | Annual total household income | $40,001 - $70,000 | $40,000 or less | 0.95 (0.74, 1.22) | 0.68 | 0.007 |
|  | Annual total household income | $70,001 - $105,000 | $40,000 or less | 0.75 (0.59, 0.95) | 0.02 |  |
|  | Annual total household income | >$105,001 | $40,000 or less | 0.75 (0.60, 0.94) | 0.01 |  |
|  | BMI |  | Increase of 1 kg/m2 | 1.01 (1.00, 1.02) | 0.16 |  |
|  | Currently smoking | Yes | No | 1.12 (0.87, 1.45) | 0.39 |  |
|  | Smoked leading up to pregnancy | Yes | No | 1.13 (0.95, 1.34) | 0.17 |  |
|  | Treatment group | n-3 | Control | 0.90 (0.80, 1.01) | 0.08 |  |

^*^ Effects are geometric mean ratios of phthalate concentrations unless otherwise indicated

^#^ Effects are relative risks of phthalate being detected

^†^ Global test for overall effect of annual total household income
